# Up-Regulation of *Hsa-miR-11181* in Glioblastoma Multiforme as A Regulator of *AKT* and *TGFBR1* Signalling 

**DOI:** 10.22074/cellj.2021.7734

**Published:** 2021-08-29

**Authors:** Hamed Dabiri, Bahram Mohammad Soltani, Sadat Dokanehiifard, Amin Jahanbakhshi, Mehdi Khaleghi

**Affiliations:** 1. Molecular Genetics Department, Faculty of Biological Sciences, Tarbiat Modares University, Tehran, Iran; 2. Stem Cell and Regenerative Medicine Research Centre, Iran University of Medical Sciences (IUMS), Tehran, Iran; 3. Department of Neurosurgery, Shariati Hospital, Tehran University of Medical Science (TUMS), Tehran, Iran

**Keywords:** *AKT*, *HBEGF*, *Hsa-miR-11181*, Glioblastoma, *TGFBR*

## Abstract

**Objective:**

MicroRNAs (miRNAs) are short non-coding RNAs that play a role in post-transcriptional regulation of gene expression. *Hsa-miR-11181* was originally introduced as a regulator of genes involved in some brain tumours. Due to the high expression of *Hsa-miR-11181* in limited glioblastoma brain tumours, in this study we intend to assess the expressions of *Hsa-miR-11181* and Has-miR11181-3p in brain tumour tissues and attribute new target genes to these miRNAs.

**Materials and Methods:**

In this experimental study, total RNA from brain tissue samples was extracted for real-time quantitative polymerase chain reaction (RT-qPCR) analysis after cDNA synthesis. In order to confirm a direct interaction of *Hsa-miR-11181* with two target genes, the 3ˊ UTR of *AKT2* and transforming growth factor-beta receptor 1 (*TGFBR1*) were cloned separately for assessment by the dual luciferase assay.

**Results:**

RT-qPCR analysis indicated that both *Hsa-miR-11181-5p* and *Hsa-miR-11181-3p* specifically up-regulated in higher grades of glioma tumours versus other brain tumour types. Consistently, lower expression levels of *AKT2* and TGFBR1 were detected in higher grade gliomas compared to other types of brain tumours, which was inverse to the level of expression detected for the heparin-binding EGF-like growth factor (HBEGF) gene. The results of the dual luciferase assay supported a direct interaction of *Hsa-miR-11181* with the 3ˊ UTR sequences of the *AKT2* and *TGFBR1* genes.

**Conclusion:**

Overall, our data suggest that miR-1118 is a potential molecular biomarker for discrimination of glioma brain tumours from other brain tumour types.

## Introduction

Primary brain tumours comprise a diverse group of malignancies that arise from various types of brain tissues. The most common brain tumours are gliomas that arise from glial cells (1). The incidence of some brain tumours have increased over time (2). Gliomas comprise various tumour types that are classified by the World Health Organization (WHO) into grades I to IV according to their invasive and proliferative behaviours (3). Glioblastoma multiforme (GBM, WHO grade IV) is the most invasive and the end-stage of most lower-grade gliomas (4). Although remote metastases to other organs is rarely reported, its high local recurrence rate and frequent central nervous system metastasis make GBM a formidable and most always fatal cancer (5). Despite treatment with chemotherapy and radiotherapy, the average survival is around 14 months (6). There are little known environmental factors in the aetiology of brain tumours; therefore, hereditary and genetic backgrounds are the focus of research (7). The failure of conventional treatments in GBM to achieve long-term survival has made it a suitable target for genetic research (8).

MicroRNAs (miRNAs) are endogenously expressed 18-25 nucleotide small non-coding RNA molecules that regulate gene expression via post-transcriptional modification (9). miRNAs play important roles in many cellular processes such as cell differentiation, survival, proliferation and metabolism (10). The dysregulation of certain miRNAs is associated with the formation of numerous types of human cancers (11, 12), and are defined as oncomiRs. Some miRNAs are tumour suppressors, whereas others have a dual function that depends on the specific tissue, time and dose of expression (13).

Hsa-miR-11181 (TrkC-miR1) is transcribed from 14^th^ intron of the human *TrkC* gene. The precursor of *Hsa-miR-11181* is processed into two partially complementary mature forms, located either at the 5´ or 3´ side of the stem loop. Hsa-miR-11181 has a positive regulatory role during neural differentiation of the NT2 cell line to neural-like cells. *Hsa-miR-11181-5p* (TrkC-miR1-5p) is relatively highly expressed in some brain-derived cell lines such as SKN-MC, A172, DAOY and 1321 (14). These cell lines originated from neuroblastoma, glioma, cerebellar medulloblastoma and brain astrocytoma tumour tissues, respectively (15).

In the present study, we evaluated the expression levels of *Hsa-miR-11181-5p* and *Hsa-miR-11181- 3p* in different brain tumour samples. *HBEGF* and *TGFBR1 *genes encode growth factor and growth factor receptors, respectively, that have neuroprotective, anti-inflammatory, proliferative and differentiation roles in neural and glial brain cells (16, 17). Also, the *AKT2* gene encodes an intermediate protein of the PI3K/ AKT signalling pathway that is necessary for synaptic plasticity and insulin-mediated glucose uptake in brain neurons and glial cells (18, 19). Therefore, the expressions of *HBEGF* and *AKT2*, as target genes of *Hsa-miR-11181-5p*, and *TGFΒR1*, as the target gene of *Hsa-miR-11181-3p*, were measured in brain tumour samples such as GBM and high-grade meningioma (grade IV). In addition, we applied a dual luciferase assay to confirm the direct targeting of these genes by *Hsa-miR-11181*.

## Materials and Methods

### Bioinformatics study

TargetScan5 (http://www.targetscan.org/vert_71/), DianamicroT and RNA-hybrid (https://bibiserv.cebitec. uni-bielefeld.de/) online tools were used for target prediction of *Hsa-miR-11181-5p* and *Hsa-miR-11181-3p*. MiRDB (http://mirdb.org/miRDB/) and miRmap (http:// mirmap.ezlab.org/) were also utilized to confirm the target predictions.

### Ethical approval 

All studies were carried out according to the latest revision of the Declaration of Helsinki (https://www.wma.net/policies-post/wma-declaration-of-helsinki-ethical-principles-for-medical-research-involving-human-subjects) and approved by the Ethics Committee of Tarbiat Modares University, Tehran, Iran (IR.MODARES.REC.1397.275). The sample collection process was confirmed and permitted by the Iran University of Medical Sciences Research Ethics Committee (IR.IUMS.REC.1398.867).

### Sample collection and RNA extraction

In this experimental study, brain tumour tissue samples were obtained from Imam, Rasoule Akram and Shariati Hospitals (Tehran, Iran). Fresh tissues were transported by liquid nitrogen and stored at -80˚C until use. Total RNA was extracted and purified from the tissue samples by TRIzol reagent according to the Invitrogen manufacturer’s protocol. RNA quantity and quality were identified by spectrophotometry and agarose gel electrophoresis, respectively. DNase treatment was conducted using RNase-free DNAaseI (Takara) at 37˚C for 30 minutes followed by heat inactivation at 75˚C for 10 minutes. 

### cDNA synthesis and real-time quantitative polymerase chain reaction

For miRNA cDNA synthesis, briefly, RNA was incubated with polyA polymerase (Takara-2180A) and ATP for 30 minutes at 37˚C. Reverse transcription was performed on the polyadenylated product. The reverse transcription (SuperScript II RT, Invitrogen, USA) reaction was accomplished by using an anchored oligo-dT primer. Table 1 lists the names and sequences of the primers used in this study. RT-qPCR was conducted in an ABI Real-Time PCR system (Applied Biosystems, USA) to evaluate the expression levels of the miRNAs and their target genes under the following conditions for 40 cycles: step 1, 95˚C for 5 seconds; step 2, 60˚C for 20 seconds; and step 3, 72˚C for 34 seconds. RT-qPCR was performed according to the guidelines and performed in duplicate. Endogenous *U48* small nucleolar RNA (*SNORD48*) and glyceraldehyde-3-phosphate dehydrogenase (*GAPDH*) were used for data analyses of the miRNAs and target expressions as the reference genes, respectively. Data were normalized using the 2^-∆∆ct^ method.

### Cell culture conditions

The HEK293T cells were cultured in DMEM-HG (Invitrogen, USA) that consisted of 10% fetal bovine serum (FBS, Gibco, USA), 100 U/ml penicillin and 100 µg/ml streptomycin. The cell lines were acquired from Pasteur Institute, Tehran, Iran.

### Dual luciferase assay

Genomic DNA, as the template, was extracted from white blood cells of a healthy person by a standard procedure. The purified DNA was used for the amplification by PCR and cloning of the region that corresponded to *Hsa-miR-11181*. The human 3´ UTR sequences of the *AKT2* and *TGFΒR1* were amplified by PCR and cloned in a psiCHECK vector downstream of the luciferase gene for the dual luciferase assay analysis with a Promega kit. Since the 3ˊ UTR of *TGFBR1 *is too long, it was cloned as two parts, named *TGFΒR1* 3ˊ UTR part 1 and part 2. *Hsa-miR-11181* and the scrambled control that contained the expression vector were used as constructed in our previous research. Sequencing of all vectors were performed for confirmation of the correct insert. 

### Transfection 

A total of 1 µg of the pEGFP-C1 vector that included the *Hsa-miR-11181* precursor and 1 µg of the 3’ UTR constructs of interest in the psiCHECK vector were mixed with Lipofectamin 2000 (Invitrogen, USA) and used for transfection of the HEK293t cell lines. After 24 hours, we used GFP microscopy (Nikon eclipse Te2000-s) to verify the successful transfection.

**Table 1:** Primer sequences used in this study


Primer name	Primer sequence (5´ to 3´)

*Hsa-miR-11181-5p*	F: GTCTGACCAACCTCCTCCC
*Hsa-miR-11181-3p*	F: AGGAGGAGGAGGTCAGG
*Hsa-miR-11181-5p and -3p*	R: AACTCAAGGTTCTTCCAGTCACG
*U48*	F: TGACCCCAGGTAACTCTGAGTGTGT
	R: AACTCAAGGTTCTTCCAGTCACG
*HBEGF*	F: ACCTATGACCACACAACCATCCTG
	R: TAGCAGTCCCCAGCCGATTCCT
*AKT2*	F: AAGAAGCTCCTGCCACCCTT
	R: CAGTAAGCCCAGGCTGTCATAG
*TGFΒR1*	F: CATTTTTCCCAAGTGCCAGT
	R: ACACCCCTAAGCATGTGGAG
*GAPDH*	F: GCCACATCGCTCAGACAC
	R: GGCAACAATATCCACTTTACCAG
*AKT2 3´UTR*	F: CTCGAGCAGCCTCCAGCCTCACCTTTG
	R: GCGGCCGCTGTGCCCACACTACGAGACC
*TGFΒR1 3´UTR part 1*	F: TCAGTCAACAGGAAGGCATCAA
	R: TTTATCAAACCATCCCTAGCCAAA
*TGFΒR1 3´UTR part 2*	F: TTAATTCCTTTTTTGGCTAGGG
	R: AACAAAAGCTTCATATCCTGGT


### Statistical analysis

Statistical analysis was carried out with GraphPad Prism 8.4.2 (GraphPad, San Diego, CA, USA). P values were evaluated by a two-sided t test and by using the repeated measures ANOVA test. P<0.05 indicated statistical significance.

## Results

### Computational prediction of the *Hsa-miR-11181* target genes 

**Fig.1:**
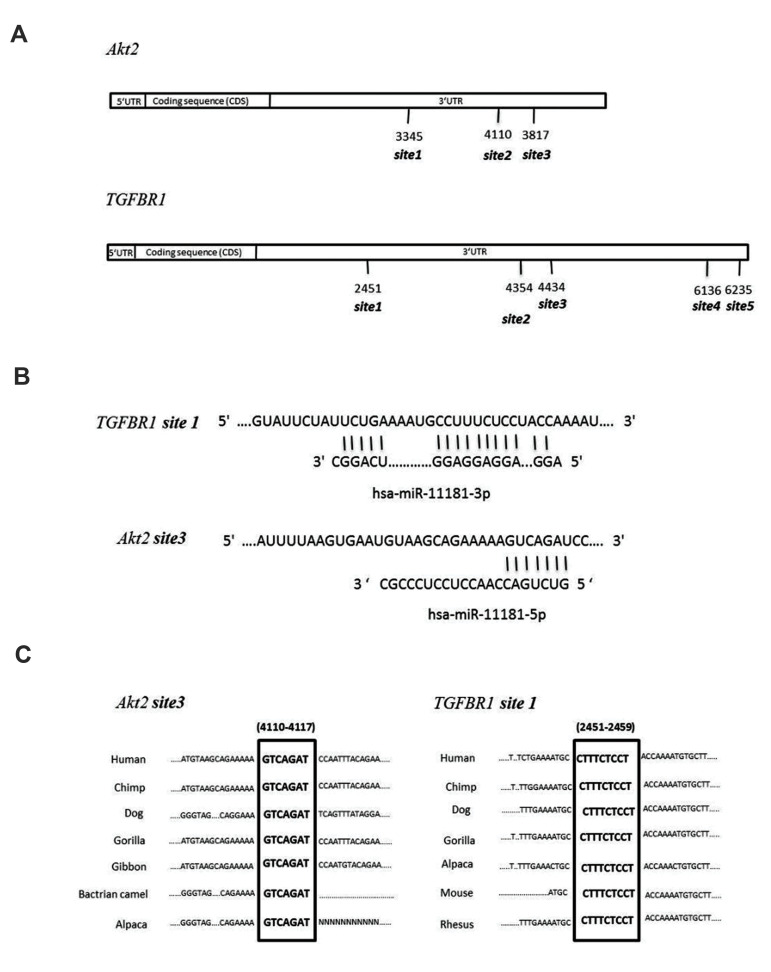
Schematic representation of the predicted miRNA recognition element (MREs) located in the 3’ UTR sequences of the *AKT2* and *TGFΒR1* genes. **A.** Shows the position of the predicted MREs located in the *AKT2* and *TGFΒR1* 3’ UTR sequences. The numbers show the first nucleotide of the MRE sequence compared to the initiation site of transcription. **B. **Pairing status of *Hsa-miR-11181* and one of its predicted MRE sequences in the *AKT2* and *TGFΒR1* 3’ UTR sequences. **C.** Shows the conservation of *Hsa-miR-11181* specific MREs in humans and other animals.

The MiRDB online tool was used to predict *Hsa-miR-11181-5p* target genes. This tool predicted approximately 209 potential targets. Then, the PANTHER and DAVID classification systems suggested the functional analyses of these predicted target genes. About 27% of the predicted targets were suggested to be involved in metabolic processes (GO: 0008152), 23% were involved in cellular processes (GO: 0009987), and the rest were involved in diverse processes that included response to stimuli, locomotion, the immune system, and biological adhesion. *AKT2*, which is involved in crucial cell signalling, was chosen for further analysis. Consistently, RNAhybrid software predicted multiple binding sites or miRNA recognition element (MREs) for *Hsa-miR-11181-5p* within the 3´ UTR sequence of the *AKT2* gene. The 3´ UTR sequence of *TGFΒR1* was predicted to be targeted by *Hsa-miR-11181-3p* and it contained multiple MREs (Fig .1).

### *Hsa-miR-11181-5p* expression status in human brain tumours

**Fig.2:**
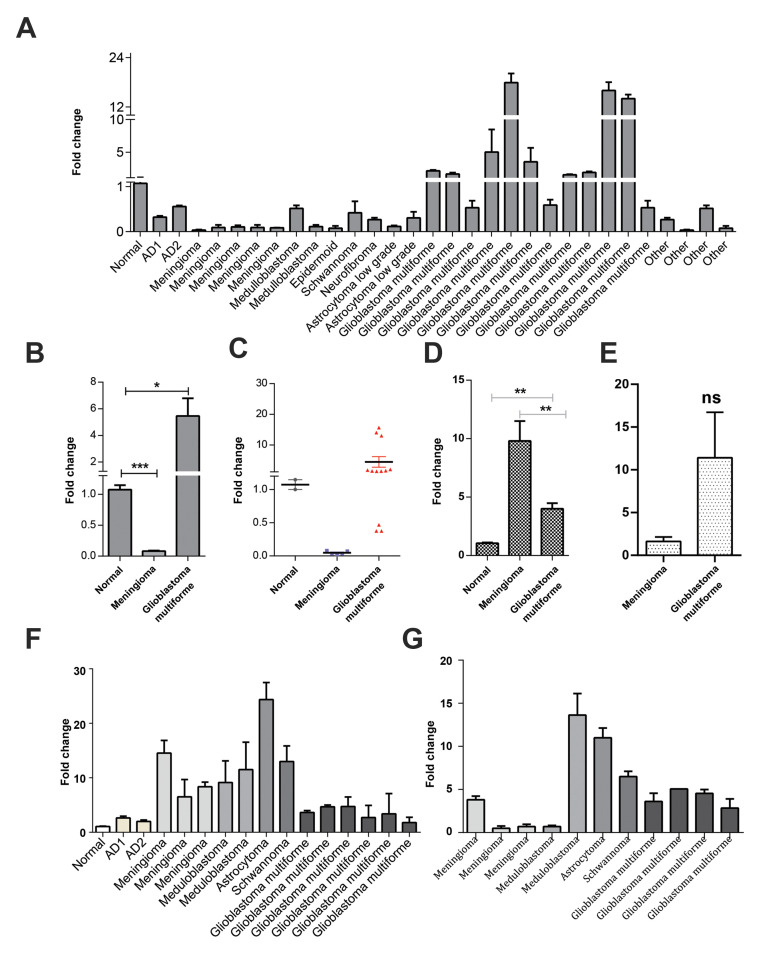
Implication of *Hsa-miR-11181-5p* in brain tumour samples. **A. ***Hsa-miR-11181-5p* expression analysis in individual brain tumour samples. **B. ***Hsa-miR-11181-5p* expression status in glioblastoma and meningioma tumour tissues related to normal brain tissue samples. **C. **Mann-Whitney analysis indicates significant up-regulation *Hsa-miR-11181-5p* in glioblastoma tumours (about 7-fold) compared to the normal samples. However, the expression of this microRNA (miRNA) significantly decreased in the meningioma samples. **D. ***AKT2* expression analysis in meningioma and glioblastoma tumour tissues, normalized against a normal brain tissue sample. **E. ***HBEGF* expression analysis in glioblastoma tumour tissues, normalized against meningioma samples. In all parts, error bars indicate SD of the duplicate experiments. **F. ***AKT2* expression analysis in individual brain tumour samples. **G. ***HBEGF* expression analysis in individual brain tumour samples. AD; Alzheimer’s disease. *; P≤0.05, **; P≤0.01, ***; P≤0.001, and ns; Not significant.

The expression level of *Hsa-miR-11181* was analysed in the following panel of 31 normal and brain tumour tissues: normal (n=1), Alzheimer’s disease (AD) affected (n=2), GBMs (grade IV, n=11), low grade astrocytoma (grade II, n=2), meningioma (n=5), medulloblastoma (n=2), epidermoid cyst (n=1), schwannoma (n=1), neurofibroma (n=1) and brain tumours without pathological typing (n=5) (Fig .2). The normal brain tissue sample used in this study was obtained from an AD patient. RT-qPCR results indicated that *Hsa-miR-11181-5p* was highly expressed in the high-grade glioma tissues compared to normal brain tissue and other types of brain tumours (P≤0.01, Fig .2A-C). However, there was no significant difference between the non-glioma brain tumours and low-grade glioma tissue (Fig .2A). Data indicated that the *Hsa-miR-11181-5p* expression level was exceptionally low in the low-grade astrocytoma samples, which was similar to the expression in meningioma samples, compared to the high-grade glioma (GBM) samples. *Hsa-miR-11181*- 5p was moderately expressed in the medulloblastoma, schwannoma, neurofibroma and epidermoid samples (Fig .2A). On the other hand, the data showed significant down-regulation of this miRNA in meningioma samples compared to the normal brain tissue sample (Fig .2A-C).

### *HBEGF* and *AKT2* target gene expression status in brain tumours

TargetScan5 and RNA-hybrid software predicted several target genes for *Hsa-miR-11181*. In order to investigate the expression status of *HBEGF* and *AKT2*, as *Hsa-miR-11181-5p* target genes, RT-qPCR was applied for 16 normal and brain tumour samples, including 6 GBM and 5 high-grade meningioma (grade IV) samples. The results indicated that the *AKT2* gene had significantly higher expression in the GBM samples compared to the normal brain sample (Fig .2D). This data was consistent with the high expression of *Hsa-miR-11181*- 5p in GBM and low expression in the meningioma samples (Fig .2A-C). The expression level of *HBEGF* in meningioma was less than the GBM samples, but the differences were not significant (Fig .2E). Figure 2F and G shows the expressions status of the *AKT2* and *HBEGF* genes in individual samples.

### *Has-miR-11181-3p* and *TGFBR1 *gene expression status in brain tumour samples

**Fig.3:**
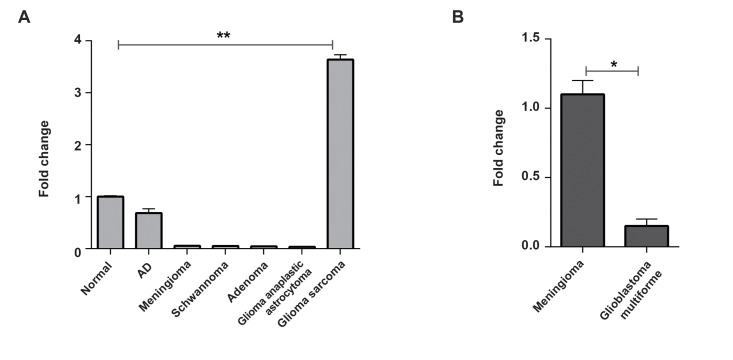
Implication of *Hsa-miR-11181-3p* in brain tumour tissues. **A.***Hsa-miR-11181-3p* expression analysis in different brain tumour samples. Data is compared to normal brain tissue samples. **B. ***TGFΒR1* expression analysis in glioblastoma tumour tissues, normalized against meningioma samples. AD; Alzheimer’s disease. *; P≤0.05 and **; P≤0.01.

*Has-miR-11181-3p* and its predicted target gene (*TGFΒR1*) expression levels were measured in 14 normal brain and brain tumour tissues, including meningioma, glioma, schwannoma, and adenoma samples (Fig .3) and AD. The expression level of *Hsa-miR-11181-3p* in the tumour samples was normalized against a normal brain tissue sample expression level. In general, the relative expression level of *Hsa-miR-11181-3p* in glioma samples was higher than in the normal brain tissue sample (Fig .3A). This result suggested a negative correlation of expression between *Hsa-miR-11181-3p* and *TGFΒR1* (Fig.3A, B). 

### Direct interaction of *Hsa-miR-11181* with the 3′ UTR sequences of the *AKT2* and *TGFΒR1* transcripts

**Fig.4:**
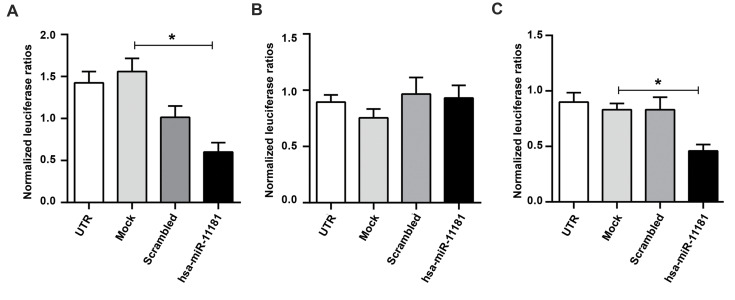
*Hsa-miR-11181* direct interaction with its predicted target genes. **A. **Dual luciferase assay supports the direct interaction of *Hsa-miR-11181* with the *TGFΒR1*, 3’ UTR sequence (part 2). **B. **Dual luciferase assay indicates no direct interaction of *Hsa-miR-11181* with the *TGFΒR1*, 3’ UTR sequence (part 1). **C.** Dual luciferase assay supports the direct action of *Hsa-miR-11181* with the *AKT2*, 3’ UTR sequence. *; P≤0.05.

The dual luciferase reporter assay was performed to investigate direct interactions of *Hsa-miR-11181-5p* and *Hsa-miR-11181-3p* with the 3′ UTR sequences of the *AKT2* and *TGFΒR1* genes, respectively. Regarding the fusion of the 3′ UTR sequences of the target genes following the luciferase reporter transcript, overexpression of miRNA leads to down-regulation of the reporter luciferase protein and a reduction in signal intensity. Therefore, this method can indicate direct interaction of the miRNA and the target transcript. In this case, when luciferase ORF was fused to the 3ˊ UTR sequences of *AKT2* or *TGFBR1 *(part 2) in the related vector and co-transfected with the vector for overexpression of *Hsa-miR-11181*, we observed a significant decrease in luciferase activity (Fig .4).

## Discussion

Up-regulation of *Hsa-miR-11181-5p* was previously reported in developing neural cells as well as in a few number of brain solid tumours, including GBM, compared to high-grade meningioma (grade IV) samples (14). Our present data also revealed an up-regulation of *Hsa-miR-11181-5p* in glioblastoma brain tissues. Most of the *Hsa-miR-11181-5p* potential target genes were predicted to be involved in metabolic and cellular processes. *Hsa-miR-11181-5p* may reprogram cellular metabolic processes that are needed for cancer progression via targeting metabolic genes. 

Bioinformatics analysis predicted that *TGFBR1*, *HBEGF* and *AKT2* are *Hsa-miR-11181* target genes. Therefore, RT-qPCR results indicated that the *AKT2* gene significantly down-regulated in the glioblastoma tumour samples in accordance with high expression levels of *Hsa-miR-11181-5p* in the glioblastoma tumours. Also, *Hsa-miR-11181-5p* expression was elevated in comparison to the normal brain sample. In meningioma samples, the expression of *Hsa-miR-11181-5p* was lower and *AKT2* was higher than the normal control, which was the opposite of glioblastoma. Therefore, the differential expression of *Hsa-miR-11181-5p* between glioblastoma and meningioma samples was remarkable. Dual luciferase assay results supported the finding that *AKT2* is targeted by *Hsa-miR-11181-5p*. The AKT protein family consists of highly homologous kinases, which are essential mediators of the PTEN/PI3K pathway, and are deregulated in many prevalent cancers in humans (20, 21). It has been reported that the AKT1 protein and its mRNA levels are similar in glioma and normal control tissues. However, there is an increase in the protein and mRNA levels of *AKT2* with the pathological grade of malignancy, whereas there is a decrease in *AKT3* mRNA and protein expressions (22). Additionally, high *AKT2* levels indicate a higher grade of meningioma and *AKT2* may play an important role in the growth of meningiomas (23). The mentioned studies are in accordance with the high expression level of *AKT2* in meningioma brain tumour samples compared to the normal brain tissue. Down-regulation of *AKT2* in U87MG, T98G and TGB cells resulted in a reduced apoptosis rate (22), which confirmed the lower expression of *AKT2* that we detected in high-grade glioblastoma (data not shown).

*HBEGF* is a confirmed target for *Hsa-miR-11181-5p* (14). However, RT-qPCR results indicated no significant transcript level changes in *HBEGF* in glioblastoma tumours compared to meningioma tissues. This might indicate that *Hsa-miR-11181-5p* affects HBEGF protein production via inhibition of translation (24, 25). On the other hand, *HBEGF* is a member of the epidermal growth factor (EGF) family that which binds to the EGF receptor employing mitogenic activity for several types of cells. The results of recent studies indicate that *HBEGF* participates in neuronal survival and proliferation of glial/ stem cells (26). It has been suggested that *HBEGF* can be a substitute for foetal calf serum (FCS) in some neuron cell cultures (27). Glioblastoma brain tumours originate from glial cells (22). Interestingly, our results indicated that GBM samples have higher levels of *HBEGF* compared to meningioma samples. Although there is no negative correlation between *HBEGF* and *Hsa-miR-11181-5p* expression in brain tumours, our results agree with the tumorigenic function of *HBEGF* in glioblastoma brain tumours.

It has been reported that the *Hsa-miR-11181-3p* (TrkC-miR1-3p) expression level does not significantly change during the NT2 cell neural differentiation. The results of research show up-regulation of *Hsa-miR-11181-3p* in glioma brain tumor samples, in comparison to meningioma (14). RT-qPCR results indicated up-regulation of *Hsa-miR-11181-3p* in glioblastoma brain tumours compared to both the meningioma samples and the normal brain tissue sample. This finding shows that *Hsa-miR-11181-3p* has a probable oncogenic role in glioblastoma. RT-qPCR results showed that *TGFΒR1*, as the *Hsa-miR-11181-3p* predicted target gene, was significantly down-regulated in glioblastoma samples compared to meningioma tumour samples. 

Dual luciferase assay results supported a direct interaction of *Hsa-miR-11181-3p* with *TGFΒR1*, which suggests that this gene is a actual target for *Hsa-miR-11181-3p*. In normal cells, *TGFB *acts as a tumour suppressor by inhibiting cell growth, stimulating cellular differentiation, and/or inducing apoptosis in a context-dependent manner. The current findings suggest that a constitutive decrease in *TGFΒR1* signalling is a strong modifier of cancer susceptibility and progression (28). Here, we have shown that *TGFΒR1* significantly down-regulated in glioblastoma samples and had a negative correlation with *Hsa-miR-11181-3p* expression. 

## Conclusion

We documented the up-regulation of two neuron related miRNAs, *Hsa-miR-11181-5p* and *Hsa-miR-11181-3p*, in high-grade glioblastoma brain tumours. Our data illustrated a significant up-regulation of *Hsa-miR-11181*- 5p in glioblastoma and down-regulation high-grade meningioma (grade IV) brain tumours. Therefore, the differential expressions of *Hsa-miR-11181-5p* and *Hsa-miR-11181-3p* were remarkable between high-grade glioblastoma and meningioma tissue samples. We also showed that miRNAs contribute to regulating the AKT and TGFB signalling pathway by targeting the *AKT2* and *TGFBR1 *genes in glioblastoma tissues. We observed down-regulation of *AKT2* in high-grade glioblastoma tissues, which confirmed the role of *Hsa-miR-11181* in the AKT2 signalling pathway. Our results indicate that the high expression level of *Hsa-miR-11181* could be applied as a potential biomarker for glioblastoma cancer detection or brain tumour categorization.
